# Finerenone: Who should prescribe it for CKD? The physician associate’s perspective

**DOI:** 10.1007/s40620-024-02015-5

**Published:** 2024-07-03

**Authors:** Becky M. Ness, Heidi Webb

**Affiliations:** 1 Department of Nephrology, Mayo Clinic College of Medicine, Rochester, MN USA; 2 Bahl & Bahl Medical Associates, Pittsburgh, PA USA

**Keywords:** Physician Associates, Diabetic Kidney Disease, Primary Care Providers, Finerenone

## Abstract

Diabetic kidney disease (DKD) affects 30–40% of all patients with diabetes and contributes significantly to the cardiovascular burden of chronic kidney disease (CKD). Despite the availability of evidence-based medications like finerenone and simple screening tests such as Urinary Albumin-to-Creatinine Ratio (UACR), more resources are still needed to care for DKD patients. Physician Associates (PAs) play a crucial role in the multidisciplinary team responsible for DKD diagnosis, monitoring, and management. A nonsteroidal mineralocorticoid receptor antagonist, namely finerenone, was approved by the FDA in adults with CKD associated with type 2 diabetes to reduce the risk of renal and cardiovascular outcomes. Finerenone is considered among the pillars of care for DKD, furthermore, the addition of finerenone in combination with renin-angiotensin system inhibitors and/or other renal protective medications may offer additional benefits. Primary care providers prescribe finerenone less frequently than specialized care providers, indicating a need to empower physician associates in medication prescription and other renal protection strategies. As part of a multidisciplinary team, physician associates can play an important role in evaluating risk factors that contribute to heart disease and metabolic health. They can also monitor not only kidney function by ordering tests, such as serum creatinine and urinary albumin-to-creatinine ratio every 3–12 months, but also serum potassium levels. Additionally, physician associates can encourage patients to take responsibility for their health by regularly monitoring their blood pressure, blood glucose levels, and body weight. With early detection and management, kidney failure and cardiovascular events may be preventable. Specialized physician associates also play a significant role in the comprehensive care of DKD patients, especially in the later stages. DKD care can be hindered by numerous factors such as lack of patient engagement during counseling, cost disparities, and a complex referral system that requires multidisciplinary guidelines to improve professional communication. It is necessary to re-envision the physician associates’ role in primary care and empower them in goal-directed therapies.

**Graphical abstract:**

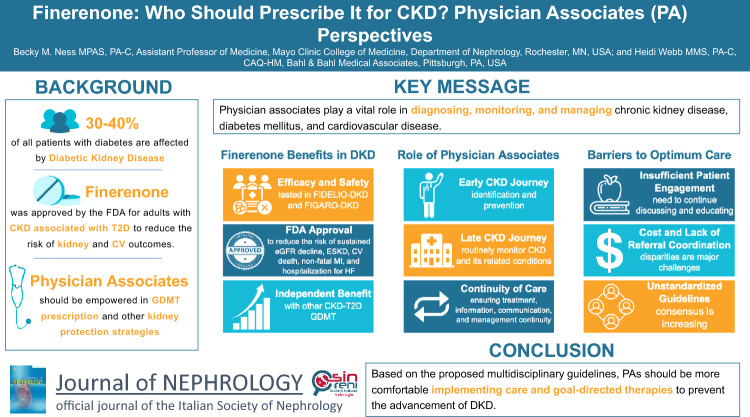

**Supplementary Information:**

The online version contains supplementary material available at 10.1007/s40620-024-02015-5.

## Introduction

According to the Kidney Disease: Improving Global Outcomes (KDIGO) guideline, Chronic Kidney Disease (CKD) is a medical condition in which the presence of a structural or functional kidney abnormality persists for three months or longer. Individuals with CKD experience a significantly higher burden of cardiovascular disease (CVD) and all-cause mortality [1]. According to a recent study that included Canada and ten European countries, the prevalence of CKD was 10%. A separate report estimated that 14% of the adult population in the United States had CKD, but only 1 in 10 Americans with CKD were aware of their condition [2]. Furthermore, approximately 30–40% of patients with all types of diabetes mellitus (DM) were estimated to also have diabetic kidney disease (DKD) [2]. However, patients with Type 1 DM are typically younger at DKD diagnosis and have fewer comorbidities, such as insulin resistance and obesity, which are commonly associated with Type 2 DM (T2DM) [3].

**Fig. 1:**
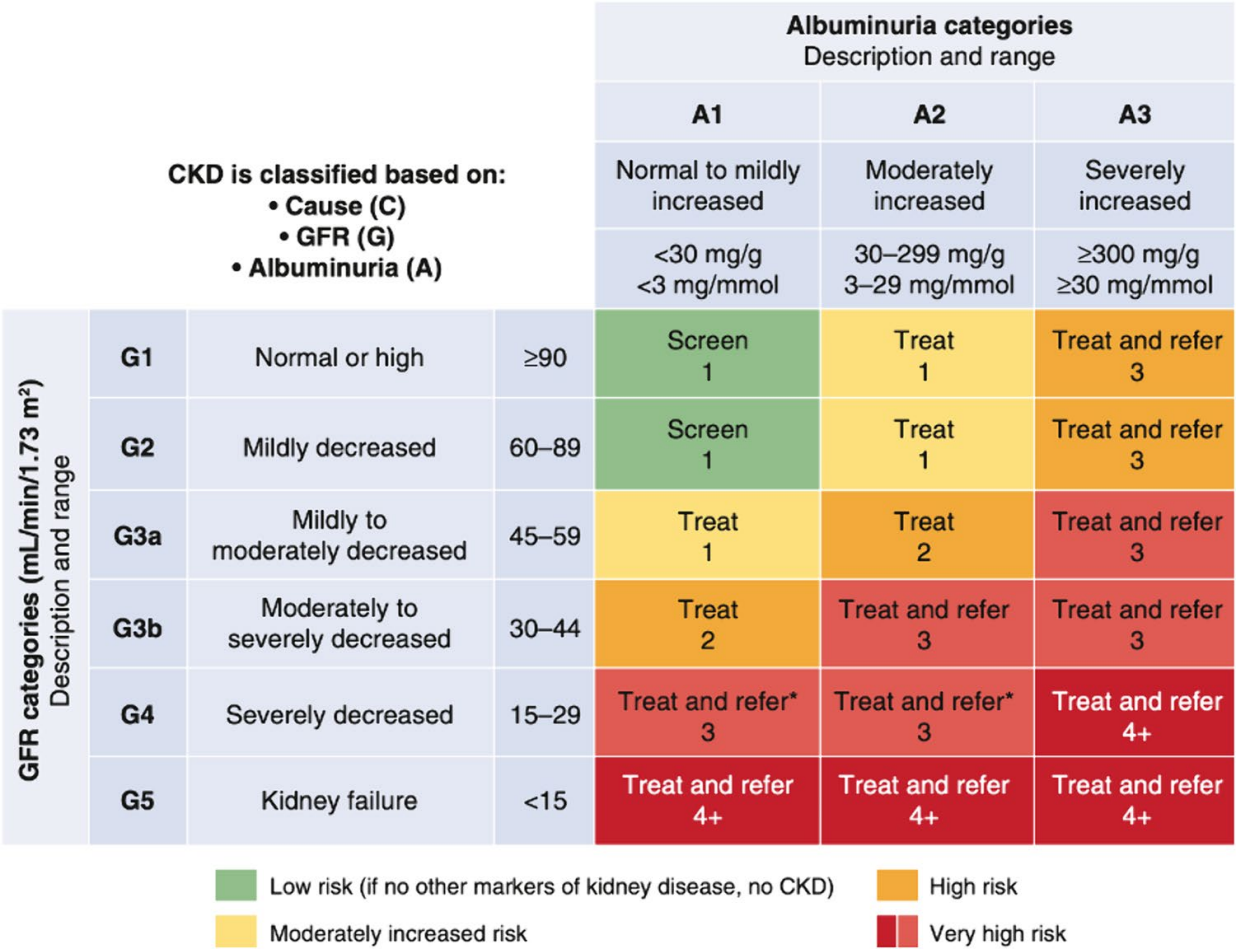
Risk of CKD progression, frequency of visits, and referral to nephrology according to glomerular filtration rate (GFR) and albuminuria. The numbers in the boxes are a guide to the frequency of screening or monitoring (number of times per year). From de Boer IH, Khunti K, Sadusky T, et al. Diabetes Management in Chronic Kidney Disease: A Consensus Report by the American Diabetes Association (ADA) and Kidney Disease: Improving Global Outcomes (KDIGO). *Diabetes Care*. Dec 1 2022;45(12):3075–3090. 10.2337/dci22-0027 [9]

Chronic kidney disease is staged based on the underlying cause, estimated glomerular filtration rate (eGFR) category (G1–G5), and albuminuria category (A1–A3). It is important to provide a complete description of the CKD stage rather than just the eGFR category [4]. Usually, serum creatinine, as the primary filtration marker, is used for estimating GFR. The CKD Epidemiology Collaboration has created a new creatinine-based eGFR equation that does not require adjustments for race as a response to the controversy of using race coefficients in clinical algorithms [5]. Urinary albumin-to-creatinine ratio (UACR) is recommended to measure albuminuria, which is more accurate than measuring urine albumin concentration alone and can be taken from a single random urine sample [6]. Both eGFR and urinary albumin-to-creatinine ratio can predict increased CVD risk [7]. Regardless of the diabetic condition, the association between impaired eGFR, albuminuria, and negative outcomes remains consistent [8]. Chronic kidney disease risk stages with reference to eGFR and albuminuria, according to KDIGO guidelines, are shown in Fig. 1. Urinary albumin-to-creatinine ratio and eGFR should be measured at least once per year in patients with low and moderate CKD risk, which include G1A1, G2A1 (green) and G1A2, G2A2, G3aA1 (yellow) [9].

Prior to the first clinical trial testing the use of renin-angiotensin system inhibitors (RASi) in 1993, there were no treatments available to stop the progression of DKD beyond glycemic and blood pressure control [10]. Substantial evidence supports that sodium-glucose cotransporter 2 inhibitors (SGLT-2i) and nonsteroidal mineralocorticoid receptor antagonists (nsMRAs) slow DKD progression when used alongside renin-angiotensin system inhibitors in T2DM patients. Moreover, the secondary outcomes of clinical trials involving glucagon-like peptide-1 receptor agonists (GLP-1RAs) have shown renal protective benefits besides their glycemic control effect [11]. However, primary studies investigating the renal benefit of Glucagon-Like Peptide-1 Receptor Agonists are ongoing and will determine the usefulness of adding a Glucagon-Like Peptide-1 Receptor Agonist to the renal protecting pillars [12]. Finerenone is a nonsteroidal mineralocorticoid receptor antagonist that has shown strong evidence in lowering the risk of CVD and DKD among a wide spectrum of patients with albuminuria [13]. The latest KDIGO guidelines have adopted a holistic approach to DKD therapies and recommended optimizing renin-angiotensin system inhibitors to the maximum toleraated dose in patients with (G1–G4, A2–A3; Fig. 1) and starting sodium-glucose cotransporter 2 inhibitors for treating patients with T2DM and eGFR ≥ 20 ml/min per 1.73 m^2^. The guidelines have also suggested starting finerenone for adults with T2DM and eGFR > 25 ml/min per 1.73 m^2^ who have (A2–A3; Fig. 1) despite the maximum tolerated dose of renin-angiotensin system inhibitors [14]. Primary care is significantly composed of physician associates (PAs) who play a vital role in diagnosing, monitoring, and managing CKD, DM, and CVD. By working collaboratively with other healthcare professionals, physician associates can take the initiative in slowing the progression of DKD, lowering mortality rates, and reducing healthcare costs [15, 16].

## Finerenone benefits in DKD

Two complementary phase III trials, FIDELIO-DKD and FIGARO-DKD, tested the efficacy and safety of finerenone and shared similar designs and endpoints. In FIDELIO-DKD, patients with stage 3–4 CKD and T2DM, who were on maximum tolerated renin-angiotensin system inhibitors, had a significantly reduced risk of the primary kidney outcomes and the key secondary cardiovascular composite outcomes when receiving finerenone [17]. Meanwhile, FIGARO-DKD showed that finerenone significantly reduced the primary cardiovascular composite outcome risk in patients with stage 2–4 CKD and moderately increased albuminuria, or in stage 1–2 CKD with severely increased albuminuria (Table 1) [18]. The FIDELITY pooled analysis, including 13,026 participants with CKD and T2DM from the FIDELIO-DKD and FIGARO-DKD trials combined, showed that finerenone reduced cardiovascular outcomes by 14% and kidney outcomes by 23% compared to placebo (Table 1) [13].

**Table 1:** Summary of the large studies showing the benefits of finerenone on cardiorenal hard outcomes

	FIDELIO-DKD	FIGARO-DKD	FIDELITY
Study design	Phase III, randomized, double-blind, placebo-controlled, multicenter clinical trial	Phase III, randomized, double-blind, placebo-controlled, multicenter clinical trial	Pooled analysis of FIDELIO-DKD and FIGARO-DKD
Sample size	(*N* = 5674)	(*N* = 7352)	(*N* = 13,026)
Publication year	2020	2021	2022
Study population	Adults with T2DM on maximum dose of RASi and Serum potassium < _4.8 mmol/L		
	G3-4^*^ CKD and A2-3 or G2^#^ and A3	G2-4^*^ CKD and A2-3 or G1-2 CKD and A3	G2-4^*^ CKD and A2-3 or G1-2 CKD and A3
Concomitant GLP-1 Ras	394 (6.9%)	550 (7.5%)	944 (7%)
Concomitant SGLT-2i	259 (4.6%)	618 (8.4%)	877 (6.7%)
Follow up duration	2.6 years	3.4 years	3.0 years
Primary outcome definition and result **[Hazard ratio (95% CI)]**	**Composite Kidney:** Renal death or Sustained eGFR < 15 mL/min/1.73 m^2^, ESKD requiring persistent dialysis or transplantation, or Sustained decline in eGFR by > 40% from baseline **0.82 (0.73–0.93)**	**Composite CV:** CV death or Non-fatal myocardial infarction or Non-fatal stroke or Hospitalization with heart failure **0.87 (0.76–0.98)**	**1-Composite Kidney:** Renal death or Sustained eGFR < 15 mL/min/1.73m^2^, ESKD requiring persistent dialysis or transplantation, or Sustained decline in eGFR by > 57% from baseline **0.77 (0.67–0.88)** **2-Composite CV:** Similar to FIGARO-DKD primary outcome **0.86 (0.78–0.95)**

G and A represent eGFR and Albuminuria KDIGO heatmap categories, respectively

*G4 with eGFR from 25 to less than 30 ml/m per 1.73 m^2^, # G2 with eGFR 75–60 ml/m per 1.73 m^2^

KDIGO, Kidney Disease: Improving Global Outcomes; CKD, Chronic Kidney Disease; CV, Cardiovascular; T2DM, Type 2 Diabetes; RASi, Renin-angiotensin system inhibitors; SGLT-2i, Sodium-Glucose Cotransporter 2 Inhibitors; GLP-1Ras, Glucagon-Like Peptide-1 Receptor Agonists; ESKD, End Stage Kidney Disease

Finerenone was first approved by the U.S. Food and Drug Administration (FDA) on July 9th, 2021, to reduce the risk of sustained eGFR decline, end-stage kidney disease (ESKD), cardiovascular death, nonfatal myocardial infarction, and hospitalization for heart failure in adults with CKD associated with T2DM [19, 20]. The National Institute for Health and Care Excellence (NICE) in the United Kingdom recommends finerenone for patients with albuminuria and eGFR ≥ 25 ml/min/1.73 m^2^ as an add-on to optimized standard care of stage 3 and 4 DKD, which includes the highest tolerated licensed doses of renin-angiotensin system inhibitors and sodium-glucose cotransporter 2 inhibitors [21]. Many healthcare providers mistakenly believe that novel glucose-lowering drugs, such as sodium-glucose cotransporter 2 inhibitors and glucagon-like peptide-1 receptor agonists, are the adequate or even ultimate solution for patients with T2DM and CKD. However, this is not accurate, considering the additional benefits of different DKD guideline-directed medical therapies achieved by addressing separate pathways [22]. The benefits of nonsteroidal mineralocorticoid receptor antagonists remain intact in patients with CKD and T2DM, despite the use of other protective medications, such as sodium-glucose cotransporter 2 inhibitors or glucagon-like peptide-1 receptor agonists [23, 24].

The large clinical trials of sodium-glucose cotransporter 2 inhibitors in DKD were started before finerenone FDA approval and included a small subgroup who were using steroidal mineralocorticoid receptor antagonists [25–27]. However, secondary analyses of randomized trials involving finerenone showed the additional benefits of introducing finerenone on top of sodium-glucose cotransporter 2 inhibitors About 7% of the FIDELITY population had concomitant use of sodium-glucose cotransporter 2 inhibitors (Table 1), and the hazard ratios for the composite endpoint of kidney disease were 0.80 (95% CI 0.69–0.92) without sodium-glucose cotransporter 2 inhibitors and 0.42 (95% CI 0.16–1.08) with sodium-glucose cotransporter 2 inhibitors [23]. The currently ongoing CONFIDENCE trial (NCT05254002) will evaluate the effectiveness of combining finerenone with empagliflozin in individuals with both CKD and T2DM, using a urinary albumin-to-creatinine ratio endpoint. The trial recruited patients with CKD stage 2–3, an eGFR of 30–90 mL/min/1.73 m^2^, a urinary albumin-to-creatinine ratio ≥ 300– < 5000 mg/g, and on maximum tolerated dose of renin-angiotensin system inhibitors. It is expected to end in mid-2024 [28].

## Role of physician associates in maximizing the benefits gained from finerenone

**Fig. 2:**
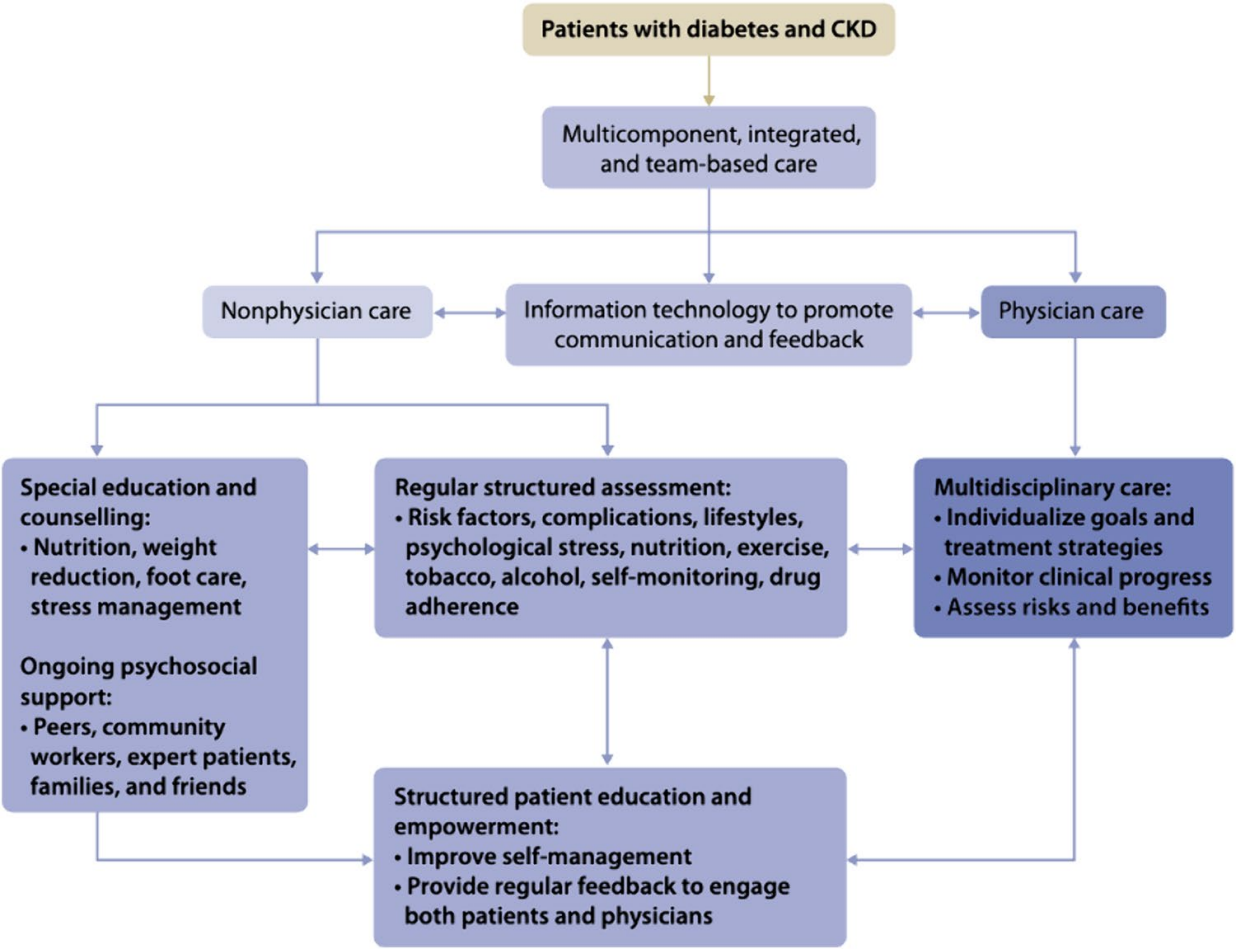
Integrated care approach suggested by KDIGO for DKD patients. From de Boer IH, Caramori ML, Chan JCN, et al. KDIGO 2020 Clinical Practice Guideline for Diabetes Management in Chronic Kidney Disease. *Kidney International*. 2020;98(4):S1–S115. 10.1016/j.kint.2020.06.019 [29]

The care of CKD patients involves various tasks that can be performed by physician associates. These tasks include assessing risk factors related to heart disease and metabolic health, monitoring kidney function through tests like serum creatinine and urinary albumin-to-creatinine ratio every 3–12 months and serum potassium, and encouraging patients to take charge of their own health through self-monitoring of blood pressure, blood glucose levels, and body weight. Furthermore, during regular check-ups, treatment targets and side effects of medications like statins, renin-angiotensin system inhibitors, sodium-glucose cotransporter 2 inhibitors, nonsteroidal mineralocorticoid receptor antagonists, and Glucagon-Like Peptide-1 Receptor Agonists are reviewed, and patients are given advice on healthy eating, exercise, and self-monitoring [29]. If non-severe hyperkalemia occurs with the prescribed medications, new potassium binders are available for continued therapy [30, 31]. Cooperation between physicians, physician associates, and other healthcare professionals, aided by technology, will enable personalized management and self-care for CKD patients. Practitioners in team-based care should use an information system with decision support to define care processes and streamline workflow for structured care (see Fig. 2) [29]. In a recent cluster randomized trial, practice facilitation programs by multidisciplinary care for primary care improved CKD and CVD outcomes [32]. Reorganizing primary care to include multidisciplinary teams can emphasize the crucial roles that physician associates are currently playing in the CKD care process and prepare the health system at the state and national level to address any potential physician shortage. It is anticipated that the United States may experience a scarcity of 37,800 to 124,000 physicians by 2034, with primary and specialty care being mainly affected [33].

However, not all primary care offices have the bandwidth to function as a multidisciplinary team. Geographic location, financial and workforce resources, reimbursement practices, and patient complexity can affect the availability and functionality of multidisciplinary teams [34]. A single institution prescription pattern showed that specialists prescribed finerenone (66%) more frequently than Primary Care Providers (PCPs). Among specialists that prescribed finerenone, 26% were in cardiology, 30% in nephrology, and 10% in endocrinology [35].

### Early CKD journey

If CKD is identified early and managed properly by non-nephrology physician associates, it may delay disease progression and avoid dialysis or transplant. In a performance improvement project, non-nephrology physician associates were provided with the Kidneys in a Box (KIB) tool. This tool focused on six modifiable factors in DKD, which are derived from the National Institutes of Health’s National Kidney Disease Education Program (NIH/ NKDEP) and includes statin use, hemoglobin A1c (HbA1c) measured within the last 6 months, urinary albumin-to-creatinine ratio measured within the last year, CKD stage, a yellow caution over-the-counter (OTC) medication list to decrease iatrogenic kidney injury, and smoking cessation [36]. Physician associates completed the program within the first 24 months, and full data were available for 213 physician associates, resulting in statistically significant behavioral changes in five of the six modifiable risk factors [36].

**Fig. 3:**
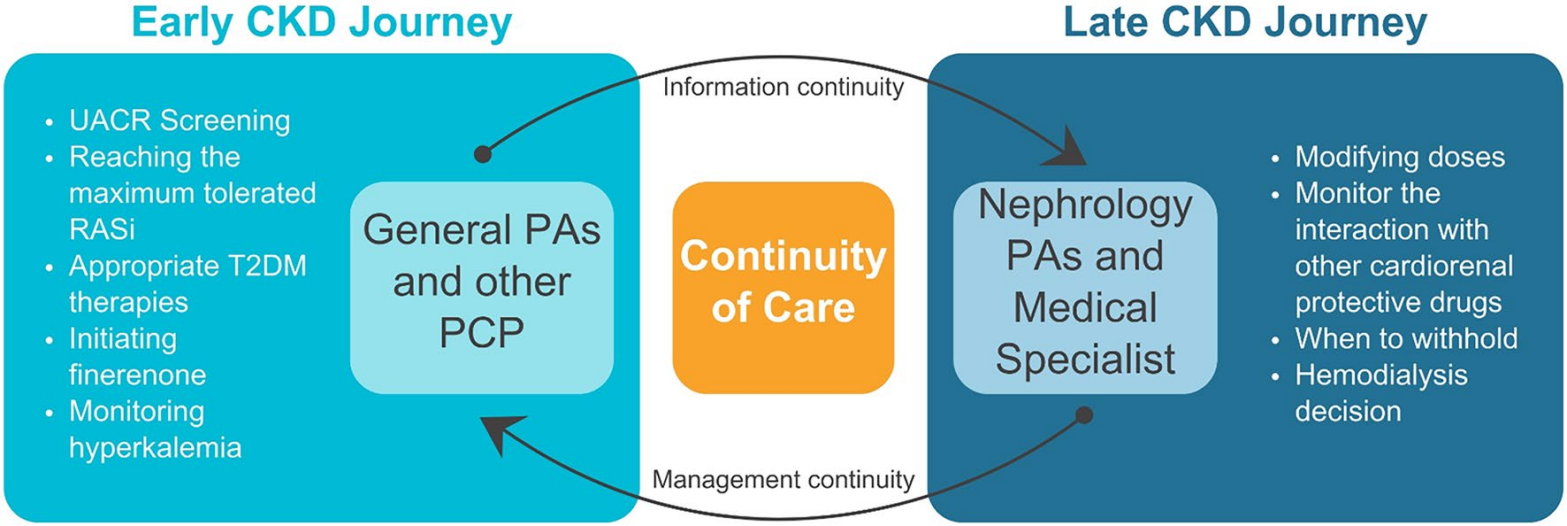
The role of physician associates in prescribing finerenone in early and late CKD journeys. CKD, chronic kidney disease; UACR, urine albumin-to-creatinine ratio; RASi, renin-angiotensin system inhibitors; T2DM, Type 2 diabetes; PAs, physician associates; PCP, primary care providers

Adjusting diabetic medications, targeting lower HbA1c levels, and detecting the stage of CKD are crucial tasks for physician associates. Measuring both eGFR and urinary albumin-to-creatinine ratio is necessary to accurately determine CKD stage. In patients with DM and urinary albumin-to-creatinine ratio levels exceeding 30 mg/g, even those who are normotensive, renin-angiotensin system inhibitors are recommended to prevent or delay the progression of DKD [37]. After reaching the maximum tolerated dose of renin-angiotensin system inhibitors, finerenone initiation is recommended if albuminuria persists [38]. Finerenone is available in 10 mg and 20 mg film-coated tablets, and the initial dose depends on the patient's eGFR. For patients with normal or slightly reduced eGFR (≥ 60 mL/min/1.73 m^2^), the recommended starting dose is 20 mg once daily. However, for those with severe renal impairment (25–59 mL/min/1.73 m^2^), the starting dose is 10 mg once a day. Patients with an eGFR below 25 mL/min/1.73 m^2^ or serum potassium concentration above 5.0 mEq/L should not initiate finerenone (Fig. 3) [39].

### Late CKD journey

Patients are diagnosed with very high CKD risk if they have G3aA3, G3bA2, G3bA3, G4A1, G4A2, G4A3, G5A1, G5A2, or G5A3 (Fig. 1) [4]. Typically, when the prevention strategies in primary care fail to halt CKD progression, patients should be referred to specialized care and renal clinics [40]. However, CKD is often not identified until late, resulting in nephrologists being the first to diagnose it. This commonly occurs when a hospitalized patient with an acute kidney injury needs a nephrology consultation, during which the nephrologist notes that the patient has had CKD for years that was never formally documented or discussed. In nephrology, historically, physician associates would only manage dialysis patients, but now they are responsible for managing complex patients in various settings such as hospitals, ICUs, offices, and dialysis units [41]. Physician associates play an important role in the team effort to aggressively treat CKD and its related conditions, such as anemia, bone and mineral disorders, electrolyte imbalances, and fluid management [42]. Furthermore, patients with late DKD stages are often prescribed multiple medications, including renin-angiotensin system inhibitors, sodium-glucose cotransporter 2 inhibitors, and finerenone, which can cause fluctuations in serum potassium levels if dosages are adjusted or stopped [39]. When starting finerenone or increasing the dosage, monitoring serum potassium and eGFR levels for at least 4 weeks is crucial. If hyperkalemia occurs, withholding or down-titrating medications that increase serum potassium levels and taking oral potassium binders can help reduce serum potassium levels and allow for the safe use of finerenone (Fig. 3) [43].

###  The importance of continuity of care in CKD

Ensuring continuity of care is crucial for high-quality healthcare, which includes information continuity and management continuity. Information continuity involves the availability and use of data from past events during current patient encounters [44]. Management continuity ensures the coherent delivery of care between different healthcare providers and institutions [44]. Higher continuity of care was associated with a lower risk of ESKD among patients with diabetic renal complications [45].

## Barriers to optimum care for CKD patients

### Insufficient patient engagement during the counseling process

The majority of patients with CKD are asymptomatic, resulting in challenges in comprehending the impact CKD has on their overall health. Chronic kidney disease as a ‘silent killer’ often presents challenges to patients in understanding its impact on overall health. Thus, physician associates play a crucial role in ongoing discussion and education of CKD patients [46]. A program designed to enhance the quality of care for patients with CKD by providing personalized educational mailings and electronic alerts to their primary care providers during office visits has been effective. The program resulted in an increase in the screening rates for urine microalbumin, leading to better identification of patients who need more intensive management [47]. In this study, almost 20% of patients did not agree with their CKD diagnosis. This is a crucial finding that highlights the importance of involving patients in the diagnosis of kidney disease, as it forms the basis for the development of effective CKD management programs.

### The cost of guideline-directed treatment

In high income countries, adoption of guideline-directed treatments for cardiorenal diseases is often impeded by high medication costs, low reimbursement rates, and the financial strain associated with clinical monitoring and medication adjustments [16]. The addition of finerenone to a hypothetical US health plan demonstrated moderately increased costs over a 3-year course. During this same time frame, minor cost savings were noted secondary to lower adverse renal and cardiovascular outcomes compared to standard care alone [48]. Furthermore, for Medicare Part D beneficiaries in 2019, the out-of-pocket costs for sodium-glucose cotransporter 2 inhibitors were at least $1,000 USD yearly, while those for Glucagon-Like Peptide-1 Receptor Agonists were over $1500 USD [49]. These costs may not be feasible for anyone, and especially burdensome on older or socially disadvantaged patients [49].

### Lack of referral coordination across different healthcare settings

Several barriers hinder the coordinated management of DKD, including the presence of multiple comorbidities, insufficient education and awareness among ethnic minorities, communication challenges between primary care providers and the multiple specialists, and a shortage of multidisciplinary care teams at clinics [50]. Although telehealth is a convenient option for referral, limited virtual physical examinations and access to technology are major concerns [51].

Telehealth contributed to mistrust among some patients. This was most consistently expressed among patients of color who preferred to see the clinician in person and read their body language. However, older adults may find it more justifiable to use due to factors such as convenience, perceived safety, engagement of care partners, and improved understanding of the patients' home environments by clinicians [51].

### Unstandardized guidelines in providing care to CKD patients

Diabetic patients with comorbid CKD and CVD could have different overlapping guideline-directed medical therapies, which represents a challenge in optimizing resources. However, the consensus between specialties guidelines is increasing [52]. The latest guidelines of different scientific societies including the European Society of Cardiology (ESC), the American Diabetes Association (ADA), the American Association of Clinical Endocrinologists, and the ADA-KDIGO consensus report recommend the use of finerenone as a treatment option for DKD [9, 53, 54]. Further research is necessary to explore implementing multidisciplinary guidelines for DKD patient referrals back to the primary care provider. Multidisciplinary guidelines will ensure consistent recommendations through incorporating the opinions of patients, primary care providers, and the related specialties [40].

## Conclusion

Re-envisioning of the physician associates’ role in primary care can facilitate adequate screening for CKD identification and provide personalized education and resources for patients with DKD. Based on the proposed multidisciplinary guidelines, physician associates should be more comfortable implementing care and goal-directed therapies to prevent the advancement of DKD.

## Supplementary Information

Below is the link to the electronic supplementary material.

Supplementary file1 (DOCX 13 KB)

## Data Availability

Data sharing not applicable to this article as no datasets were generated or analyzed during the development of this review article.

## Abbreviations

KDIGO: Kidney Disease: Improving Global Outcomes
CKD: Chronic Kidney Disease
CVD: Cardiovascular Disease
DKD: Diabetic Kidney Disease
T2DM: Type 2 Diabetes
UACR: Urinary Albumin-to-Creatinine Ratio
RASi: Renin-angiotensin system inhibitors
SGLT-2i: Sodium-Glucose Cotransporter 2 Inhibitors
nsMRA: Nonsteroidal Mineralocorticoid Receptor Antagonist
GLP-1Ras: Glucagon-Like Peptide-1 Receptor Agonists
PAs: Physician Associates
FDA: Food and Drug Administration
ESKD: End Stage Kidney Disease
NICE: The National Institute for Health and Care Excellence
HbA1c: Hemoglobin A1c
OTC: Over-The-Counter
PCP: Primary Care Providers
