# Supplementary material for: Finerenone: Who should prescribe it for CKD? The physician associate’s perspective
Source: J Nephrol. 2024 Jul 3;37(8):2161–70. doi: 10.1007/s40620-024-02015-5 (PMC11649777; doi:10.1007/s40620-024-02015-5)
Supplement: Supplementary file 1 — Supplementary file1 (DOCX 13 KB) [file 40620_2024_2015_MOESM1_ESM.docx]

**Supplementary Material**

**Plain Language Summary**

Gradual loss of kidney function (i.e., chronic kidney disease) is common among patients with abnormally high blood sugar (i.e., diabetes) and is associated with heart disease. The severity of kidney damage can be detected by measuring the creatinine level in the blood and detecting the excreted protein in urine. Several medications are now recommended by guidelines, including finerenone, to prevent the progressive loss of kidney function. However, the use of these recommended medications is influenced by multiple healthcare professionals involved in the early diagnosis, patient counseling, and monitoring of the drug.

Physician Associates (PAs) play a crucial role in providing necessary multidisciplinary care, ensuring that individuals with chronic kidney disease receive reliable and continuous care. At the primary care level, they encourage healthy lifestyle intervention to preserve kidney function and emphasize the importance of early urine screenings to detect the disease. Furthermore, PAs collaborate with specialists to adjust medication dosages according to individual needs and potential side effects.

There are key challenges that limit the PAs ability to optimally manage chronic kidney disease (CKD) associated with diabetes. These include involving patients in their care, improving how referrals are handled, cost of services, including medications, and having clear multidisciplinary guidelines to follow. By taking an integrated approach, we can ensure that PAs are able and ready to implement medical therapies directed by guidelines, like finerenone.
